# Identification of Single Nucleotide Polymorphisms in Porcine *MAOA* Gene Associated with Aggressive Behavior of Weaned Pigs after Group Mixing

**DOI:** 10.3390/ani9110952

**Published:** 2019-11-11

**Authors:** Ruonan Chen, Qingpo Chu, Chunyan Shen, Xian Tong, Siyuan Gao, Xinpeng Liu, Bo Zhou, Allan P. Schinckel

**Affiliations:** 1 College of Animal Science and Technology, Nanjing Agricultural University, Nanjing 210095, China; 2016105033@njau.edu.cn (R.C.); qpchu1990@163.com (Q.C.); 2016105082@njau.edu.cn (C.S.); 2017105081@njau.edu.cn (X.T.); 2018105082@njau.edu.cn (S.G.); 2018805122@njau.edu.cn (X.L.); 2 Department of Animal Sciences, Purdue University, West Lafayette, IN 47907-2054, USA; aschinck@purdue.edu

**Keywords:** aggressive behavior, behavioral genetics, monoamine oxidase A, swine, SNPs

## Abstract

**Simple Summary:**

Monoamine Oxidase A (*MAOA*) gene had been reported as a candidate gene of aggressive behavior in several species. In the present study, the most aggressive and docile weaned pigs in each pen after group mixing were selected to identify single nucleotide polymorphisms in porcine *MAOA* gene associated with aggressive behavior. Constructs containing variable lengths of truncated porcine *MAOA* promoter were used to determine the promoter activity by a dual luciferase reporter system. The core promoter region of porcine *MAOA* was located at −679 bp to −400 bp. A total of nine single nucleotide polymorphisms (SNPs) in the *MAOA* gene were genotyped, of which six SNPs had significant differences in allele frequency between the aggressive and docile pigs. Four linked SNPs in porcine *MAOA* gene were associated with aggressive behavior in weaned pigs after mixing, which can be used as candidate molecular markers for aggressive behavior in pigs.

**Abstract:**

Understanding the genetic background underlying the expression of behavioral traits has the potential to fasten the genetic progress for reduced aggressive behavior of pigs. The monoamine oxidase A (*MAOA*) gene is known as the “warrior” gene, as it has been previously linked to aggressive behavior in humans and livestock animals. To identify single nucleotide polymorphisms in porcine *MAOA* gene associated with aggressive behavior of pigs, a total of 500 weaned pigs were selected and mixed in 51 pens. In each pen, two aggressive and two docile pigs (a total of 204 pigs) were selected based on their composite aggressive score (CAS). Ear tissue was sampled to extract genomic DNA. Constructs containing variable lengths of truncated porcine *MAOA* promoter were used to determine the promoter activity by a dual luciferase reporter system. The core promoter region was located at −679 bp to −400 bp. A total of nine single nucleotide polymorphisms (SNPs) in *MAOA* gene were genotyped, of which six SNPs had significant differences (*p* < 0.05) in allele frequency between the aggressive and docile pigs. Linkage disequilibrium and association analyses showed that the pigs inherited the wild genotypes showed more aggressive behavior (*p* < 0.05) than pigs with the mutant genotypes of the four linked SNPs, rs321936011, rs331624976, rs346245147, and rs346324437. In addition, pigs of GCAA haplotype were more (*p* < 0.05) aggressive than the pigs with GCGA or ATGG haplotype. The construct containing the wild genotype GG of rs321936011 had lower (*p* = 0.031) promoter activity compared to the mutant genotype AA. These results suggest that the four linked SNPs in *MAOA* gene could be considered as a molecular marker for behavioral trait selection in pigs.

## 1. Introduction

It is a common procedure that piglets are regrouped after weaning to create homogenous groups for equalizing competition in commercial pig farming [1]. To reestablish a new social hierarchy, weaned pigs often show a high frequency of agonistic behavior after mixing [2]. Agonistic behavior of weaned pigs lasted approximately 4 days after mixing until a stable hierarchy had been established [3]. The most intense fighting after mixing mainly occurred within the first 24 h [4]. The agonistic behavior after mixing is an animal welfare concern because it caused skin lesions on the body [5] and decreased the growth performance in pigs [6]. To reduce agonistic behavior of pigs, a number of practical interventions have been explored [1], but no clear economically effective solution has been identified. Previous studies showed that Chinese indigenous pig breeds are more docile than European pig breeds [7]. The heritability parameters of aggressive behavioral traits indicate that the visible expression of aggression is heritable in pigs [8]. Aggressive behavior could be reduced through genetic selection [9], which has been successively used in poultry [10]. Identification of molecular genetic markers associated with agonistic behavioral traits could contribute to optimize the genetic selection.

A number of genes related to the serotonin neurotransmitter pathway, including the monoamine oxidase A and B (*MAOA* and *MAOB*), serotonin transporter (*SLC6A4*), tryptophan hydroxylases 1 and 2 (*TPH1* and *TPH2*), catechol-*O*-methyl transferase (*COMT*), and dopamine-beta-hydroxylase (*DBH*) have been linked to anxiety and stress response in pigs [11]. Neurotransmitter 5-hydroxytryptamine (*5-HT*) in the hypothalamus regulates dominance and aggression in hamsters [12]. The enzyme MAOA in the brain catalyzes the oxidative deamination of biogenic amines, including 5-hydroxytryptamine and dopamine [13]. This consequently influences aggressive behavior because of the degradation of neurotransmitters in the brain [14]. The knock-out of the *MAOA* gene in mice caused an increase in concentration of dopamine, serotonin, and norepinephrine in the brain, which consequently induced an increase in aggressive behavior in males [15,16]. This suggests that the activity of the *MAOA* enzyme is negatively related with aggressive behavior in male mice.

Variations in the *MAOA* gene in human and mouse have been associated with aggressive behavior traits [15,17]. In human, a 30 bp variable number of tandem repeats (VNTR) is located in the promoter region of *MAOA* gene and down-regulates the transcriptional activity [18]. The SNP rs6323 (T941G) is located in the exon 8 of the *MAOA* gene, its TG or GG genotype codes a higher activity of the enzyme than its TT genotype [19]. Association between the T941G *MAOA* polymorphism and aggressive behavior in adolescents has been reported [19]. In addition, another SNP rs1465108 in the *MAOA* gene has been associated with aggressive behavior in humans [20].

Our previous study found that Chinese indigenous pigs are more docile than European pigs, and the genetic polymorphism has considerable difference in neurotransmitter-related genes between pig breeds [7]. The Suhuai pig breed, a composite breed containing 25% Chinese indigenous Huai pig and 75% European Yorkshire pig [21], is a genetic resource to identify the genetic markers associated with aggressive behavior in pigs.

Therefore, we hypothesized that some of single nucleotide polymorphisms (SNPs) of porcine *MAOA* gene could affect its expression level or function, which influences aggressive behavior of pigs after group mixing. The objective of this study is to identify the SNPs related to aggression in porcine *MAOA* gene through association between polymorphism of *MAOA* gene and the indicators of aggressive behavior in weaned pigs after mixing.

## 2. Materials and Methods

### 2.1. Animals and Housing

This study was approved by the Animal Care and Use Committee of Nanjing Agricultural University (SYXK2017-0007). A total of 500 weaned pigs (268 barrows and 232 gilts) from 76 litters of multiparous sows (parity 2–5) were selected in the Huaiyin Pig Breeding farm (Huaian, Jiangsu, China). Piglets were weaned at 35 d of age (body weight 13.85 ± 0.31 kg) and moved to empty pens with their original littermates in a nursery room before mixing. First, pigs were blocked by sex and body weight. Nine or ten weaned pigs with the same sex and similar body weight from different litters were mixed in pens of dimension 2.5 m × 2.2 m. The pens were equipped with slatted floors, stainless-steel vibratory feeders and nipple drinkers to allow ad libitum access to feed and water. All experimental pigs were weighed at 24 h before mixing and at 72 h after mixing. Ear tissue of each weaned pig was collected for DNA extracting.

### 2.2. Behavioral Assessment

Pig behavior was recorded for 72 h continuously after mixing using a digital video recording system (Hikvision DS-2CE56C2P-IT3 3.6 mm; Hikvision network hard disk video recorder DS-7808HW-E1/M; Hikvision Digital Technology Co. Ltd., Hangzhou, China). The pigs in each pen were individually identified using a spray paint (7CF, Shenzhen Zhaoxin Energy Co., Ltd., Shenzhen, China) on the back and both sides of each pig’s body. Aggressive behavior score of each pig was assessed by observing the video and recording the frequency and duration of fighting behaviors of each pig within 36 h after mixing in each pen. For each aggressive event, the initiator of the fight, the frequency and duration of active attack, being bullied and standoff behaviors were recorded (Table 1). A fighting behavior was defined as a fight or a displacement event when the physical contact of two individuals lasted for more than 3 s, the intervening period between fighting behaviors was at least 8 s [22]. In a fight, biting, pushing, and chasing was identified as the active attack behavior [23]. When the recipient pig suffers from biting and head-knocking performed by the aggressive pig and the recipient moves away without retaliation, it was identified as being bullied [24]. If the two pigs stood side by side, shoulder to shoulder, and one pig threw his head to the head or neck of the other pig, there was an aggressive interaction with no dominance sign produced by either pair member at any time, it was defined as a standoff event [25]. When a pig showed a submissive behavior, such as stopping its fighting, turning away from an attack, trying to flee or was displaced from the location, it was defined as a loser [26], and the other pig in the fight was defined as a winner [27]. If there was no clear outcome, the fight was designated as a draw [22].

### 2.3. Aggression Assessment

Composite aggressive score (CAS) was calculated to express the aggression of each pig adapted from [28]:

CAS = (Frequency of active attacks) + 0.2 * (Duration of active attacks [s])

The two most aggressive pigs and the two most docile pigs were selected by the CAS in each pen. A total of 102 aggressive and 102 docile pigs were selected from 500 pigs for the SNP association analyses.

### 2.4. Genetic Polymorphism Analyses of MAOA Gene

#### 2.4.1. Promoter Prediction of the Porcine MAOA Gene

The promoter region of the porcine *MAOA* gene (ENSSSCG00000012257) was predicted by Promoter Scan (www-bimas.cit.nih.gov/molbio/proscan), Promoter 2.0 (www.cbs.dtu.dk/services/Promoter), and Neural Network Promoter Prediction (www.fruitfly.org/seq_tools/promoter.html). Putative transcriptional binding start sites of the *MAOA* gene were predicted by AliBaba 2.1 (http://gene-regulation.com/pub/programs/alibaba2/index.html). Methylation sites were predicted by Meth-Primer 2.0 (www.urogene.org/methprimer) and microRNA binding sites in 3′-untranslated region (UTR) region of *MAOA* gene were predicted by Target Scan 7.2 (www.targetscan.org/vert_72).

#### 2.4.2. Plasmid Construction

The promoter region of the porcine *MAOA* gene was amplified by PCR using Taq DNA polymerase (Takara, Dalian, China). Subsequently, constructs containing variable length of truncated pig *MAOA* promoter were individually amplified using different forward primers and a common reverse primer. For plasmid ligation, the forward primers and reverse primer contained SacⅠ and XhoⅠ recognition sequences, respectively (*MAOA*-P1, −78/+138; *MAOA*-P2, −400/+138; *MAOA*-P3, −679/+138; *MAOA*-P4, −938/+138; *MAOA*-P5, −1534/+138; *MAOA*-P6, −2148/+138; Supplemental Table S1). The amplified fragments were then inserted into the multiple cloning site of the pGL3-basic vector to generate luciferase reporter constructs. Specific regions containing SNP rs321936011 were amplified using *MAOA*-P7 primers, the forward primers and reverse primer contained SacⅠ and HindⅢ recognition sequences (Supplemental Table S1). All plasmids were sequenced to confirm proper insertion prior to transfection experiments.

#### 2.4.3. Cell Culture, Cell Transfection, and Luciferase Assays

Analysis of promoter activity was based upon human renal epithelial cell-293T cell (ATCC^®^ACS-4004™). Transfections in 293T cells were performed using Lipofectamine 2000 (Invitrogen, Carlsbad, California). Cells were plated in 12-well plates. The following day, the *MAOA* promoter-luciferase construct was co-transfected with plasmid pRL-TK (the herpes simplex virus thymidine kinase promoter fused upstream to the Renilla luciferase gene, which is used as an internal control; Promega, Wisconsin, USA) into the 293T cells. Controls were the pGL3-basic and pGL3-control luciferase reporter gene vector instead of the *MAOA* promoter luciferase construct. After 24 h, cells were harvested with luciferase assay lysis buffer (Promega, Wisconsin, USA). The cell lysates were assayed for luciferase activity using the Promega Dual Luciferase Assay system. All reactions were replicated nine times (n = 9).

#### 2.4.4. Potential SNP Identification

Total DNA was extracted from ear tissue of pigs by standard phenol/chloroform method (Roche, Beijing, China). The specific primers (Supplemental Table S1) were designed with Primer-BLAST in NCBI (www.ncbi.nlm.nih.gov) to amplify 5′-UTR, 16 exons and 3′-UTR of the porcine *MAOA* gene. PCR reactions were performed using rTaq and LATaq Master Mix (Takara, Dalian, China). The amplified PCR products were sequenced, and multiple sequence splice and alignments were performed to analyze the SNPs between aggressive and docile pig groups using the software DNAMAN 8.0 (www.lynnon.com/index.html) and Chromas 2.6.4 (omictools.com/chromas-tool).

### 2.5. Data Analyses

The data of behavior was analyzed using PROC GLIMMIX procedure in SAS 9.4 (SAS Institute Inc, Cary, NC, USA). The fixed effects were sex, parity, and group which the pigs split into two equal sized groups of the two most docile and two most aggressive pigs in each pen of 9 or 10 pigs, and pen as a random effect. The relative fluorescence activity value was normalized by positive control pGL3-control. Data of luciferase activity were analyzed by the one-way ANOVA analysis. A canonical correlation analysis was performed between the score of aggressive behavior indicators and the genotypes of SNPs. To compare gene frequencies between aggressive and docile pigs, a chi-square analysis was performed. Aggressive behavior indicators of pigs with different genotypes/haplotypes of the four linked SNPs were determined using PROC GLIMMIX procedure with a model option DIST = EXPO in SAS. The fixed effects were sex, genotype or haplotype, and pen as a random effect. The results are presented as mean ± SEM and *p*-value < 0.05 was considered significant. Linkage disequilibrium was calculated using software HAPLOVIEW 4.2 (www.broadinstitute.org/haploview/haploview).

## 3. Results

### 3.1. The Difference of Behavioral Indicators between Aggressive and Docile Weaned Pigs after Mixing

**Figure 1:**
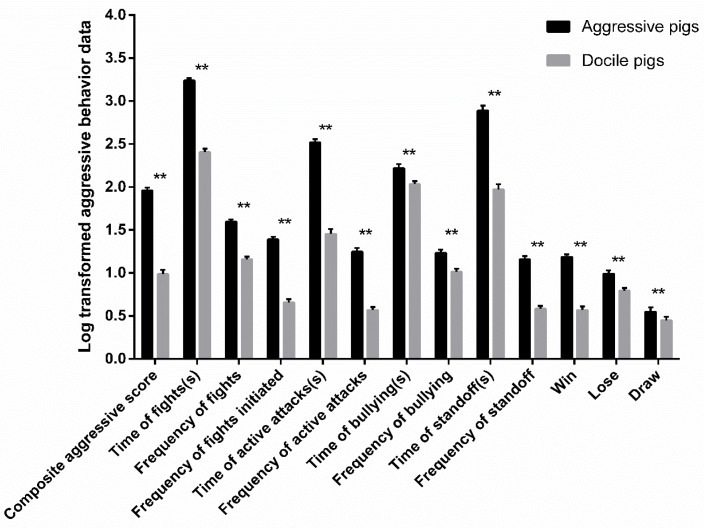
The log transformed aggressive behavioral indicators within 36 h after mixing between aggressive pigs (n = 102) and docile pigs (n = 102). ** *p* < 0.01.

A total of 204 aggressive or docile pigs were selected based on their composite aggressive score (CAS) (118.72 ± 8.26 vs. 14.53 ± 2.04; F (1189) = 121.222, *p* = 0.000) (Figure 1). The aggressive pigs showed more fights, including active attack, standoff behavior, and initiated more fights (*p* < 0.01).

### 3.2. Prediction and Identification of the Core Promoter in Porcine MAOA Gene

#### 3.2.1. *MAOA* Promoter Prediction

Four promoter regions (−1159/−1109, −567/−517, −546/−496, and −735/−485), and two transcription initiation sites (−1800 and −400) of the porcine *MAOA* gene were identified by the Promoter Scan, Promoter 2.0 and Neural Network Promoter Prediction tools. Six CpG island signals (−1762 to −1647, −1588 to −1482, −1470 to −585, −692 to −585, −423 to −215, −156 to +59 bp) were found in the promoter region. Sequence analysis of the porcine *MAOA* promoter segments revealed that the 5′-UTR region harbored potential binding sites for multiple transcription factors including AP-1, Sp1, MEF2C, MEF2A, TBP, Sox3, GATA3, CEBPA, Sox17, FOXL1, POU2F2, Foxd3, TCF7L2, SREBF1, and Nkx2-5.

#### 3.2.2. *MAOA* Promoter Identification

**Figure 2:**
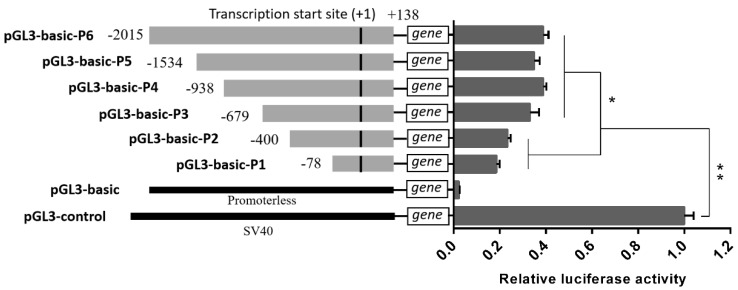
Luciferase assays for porcine *MAOA* promoter activity. Six luciferase reporter plasmids expressing successive truncations of the *MAOA* promoter sequence were constructed and transfected into 293T cells. The resulting firefly luciferase activity was normalized to Renilla luciferase activity and the relative values were presented as fold induction over the activity of the pGL3-basic vector. The basic activity value of negative control pGL3-control was set as 1. The location of each 5′-deletion sequence was indicated at the left of each bar. The relative luciferase activity values represent the mean ± SEM of three independent experiments. Statistical differences in luciferase activity were assessed using the one-way ANOVA analysis, * *p* < 0.05, ** *p* < 0.01.

The Luciferase activity of the six *MAOA* promoter fragments was greater than the pGL3-basic groups (*p* < 0.01) and less than the pGL3-control groups (*p* < 0.01). The luciferase activity of constructs P6 (−2015/+138), P5 (−1534/+138), P4 (−938/+138), and P3 (−679/+138) was greater than those of constructs P2 (−400/+138) and P1 (−78/+138) (F (7, 74) = 159.617, *p* = 0.018) (Figure 2). These results have shown that the core promoter region of the *MAOA* gene is located between −679 bp and −400 bp.

### 3.3. Screening of SNPs in the Porcine MAOA Gene Associated with Aggression

#### 3.3.1. *MAOA* Gene Polymorphism and Aggressive Behavior and Linkage Disequilibrium (LD) Analyses

**Figure 3:**
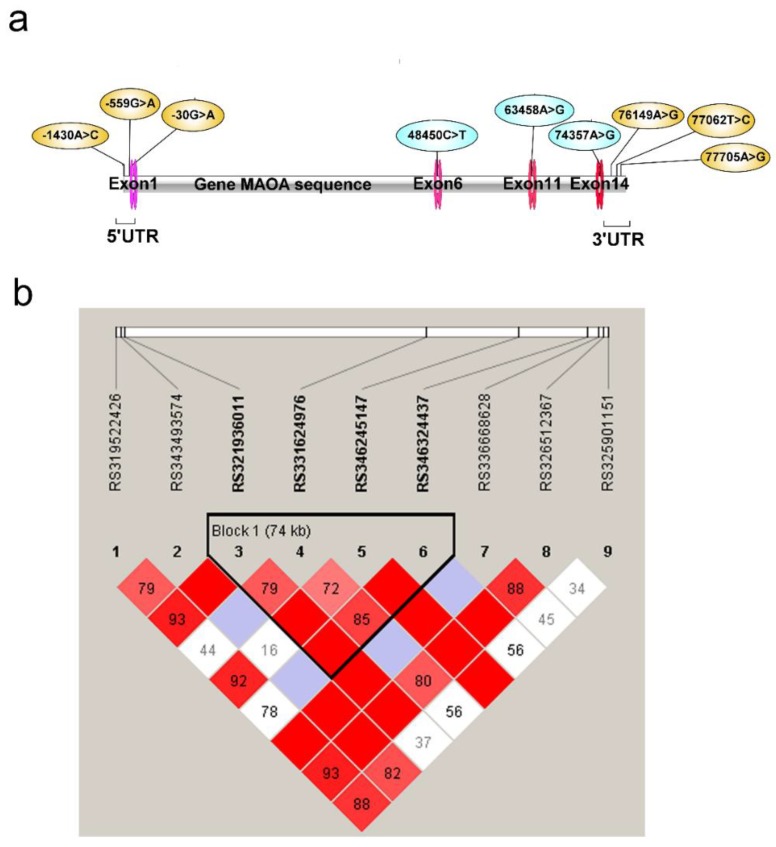
The linkage disequilibrium analysis of nine SNPs in the *MAOA* gene. (**a**) The position of nine SNPs in pig *MAOA* gene. (**b**) Linkage disequilibrium analysis of the nine potential SNPs. Four (rs321936011, rs331624976, rs346245147, and rs346324437) of nine potential SNPs showed linkage inheritance resulting in five haplotypes (GCAA, ACGA, GCGA, ATGG, and ACGG/GTAA).

To detect the porcine *MAOA* gene polymorphisms, three SNPs, rs319522426 (−1430 A or C), rs343493574 (−559 G or A), and rs321936011 (−30 G or A) in the 5′-UTR; three SNPs rs336668628 (+76149 A or G), rs326512367 (+77062 T or C), and rs325901151 (+77705 A or G) in 3′-UTR; three SNPs rs331624976 (+48450 C or T), rs346245147 (+63458 A or G), and rs346324437 (+74357 A or G) in the introns were identified in the pigs. Genotypic distribution of rs331624976 and rs325901151 deviated from Hardy–Weinberg equilibrium (HW *p* < 0.05 or HW *p* = 1), whereas the other seven SNPs were in accordance with Hardy–Weinberg equilibrium (Table 2). Except for rs343493574, rs336668628, and rs325901151, the other 6 SNPs showed significant difference (*p* < 0.05) in allele frequencies between aggressive and docile pigs by chi-square analyses (Table 2). Four SNPs rs321936011, rs331624976, rs346245147, and rs346324437 made up a linkage inheritance and formed five haplotypes (GCAA, ACGA, GCGA, ATGG, and ACGG/GTAA) (Figure 3).

#### 3.3.2. Differences of Behavioral Indicators between Genotypes/Haplotypes of Four Linked SNPs

Pigs with the wild genotype (major genotype) GG of rs321936011 had greater CAS, duration of fights, duration of active attacks, and duration of standoff than those with the mutant genotype AA (minor genotype) (*p* < 0.05, Table 3). Pigs with the genotype CC of rs331624976 had greater CAS, duration of fights, duration and frequency of active attacks, duration and frequency of standoff than that of pigs with the genotype TT (*p* < 0.05). Pigs with the genotype AA of rs346245147 had greater CAS, duration and frequency of fights and standoff than pigs with genotype GG (*p* < 0.05). Pigs with genotype AA of rs346324437 had greater CAS, duration and frequency of fights, active attacks, and standoff than pigs with genotype GG (*p* < 0.05). It is interesting that pigs with major genotypes of four linked SNPs in *MAOA* gene were more aggressive than pigs with minor genotypes.

Further association analyses on haplotypes showed that pigs with the haplotype GCAA had greater CAS than those with the haplotype GCGA or ATGG (*p* < 0.05). More specifically, GCAA haplotype pigs had more duration and frequency of fights, active attacks, and standoff (*p* < 0.05; Table 4). In addition, the number of barrows were greater than the number of gilts in the major genotypes of four linked SNPs and haplotype GCAA (*p* < 0.01; Supplemental Tables S2 and S3).

### 3.4. Promoter Activity Analyses of Porcine MAOA Gene

**Figure 4:**
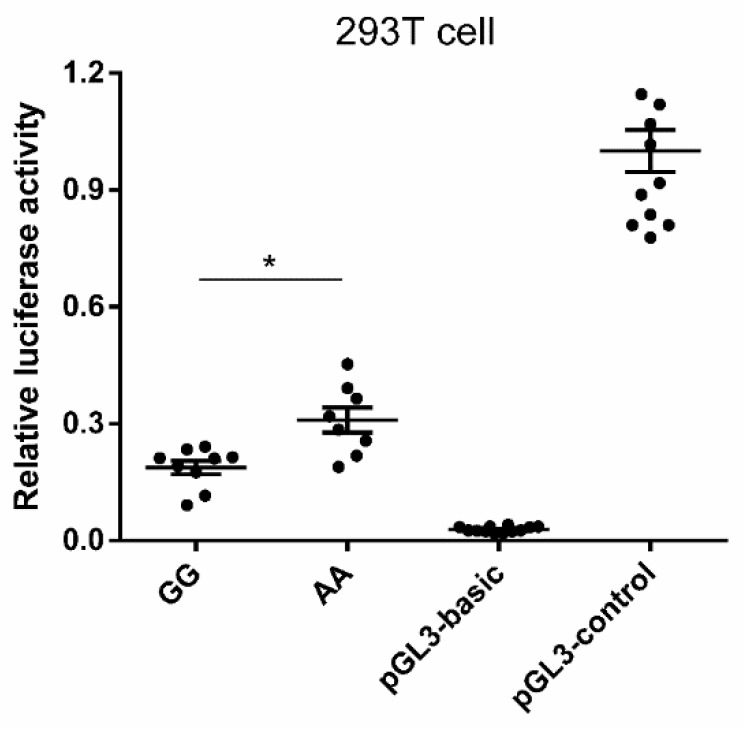
Luciferase reporter gene assays of porcine *MAOA* alleles containing rs321936011 (−30 G or A). Two genotype luciferase reporter vectors of the *MAOA* −480 to +154 bp sequence were constructed and transfected into 293T cells. The firefly luciferase activity was normalized to Renilla luciferase activity and the relative values were presented as fold induction over the activity of the pGL3-basic vector. The basic activity value of positive control pGL3-control was set as 1. The location of base is 30 G or A. The relative luciferase activity values represent the mean ± SEM of three independent experiments. Statistical differences in luciferase activity were assessed using the one-way ANOVA analysis, * *p* < 0.05.

One of the six candidate SNPs, −30 G > A, was located downstream of the core promoter region of *MAOA*. It was also linked with the other three significant SNPs, which were located in the intron and splice regions. Promoter activity of different genotypes of rs321936011 in the porcine *MAOA* gene was analyzed using the dual-luciferase reporter assay system (www.promega.com.cn). Our bioinformatics analysis showed that the mutation of this SNP would result in the variation of several transcription factors, such as the disappearance of EGR1 and FOXC1 and the appearance of FOXA1 and ZNF354C (Supplemental Table S4). As shown in Figure 4, luciferase reporter constructs of GG genotype had lower (F (3, 41) = 168.635, *p* = 0.031) luciferase activities than those of AA genotype. The luciferase activities of constructs with GG and AA genotypes were greater than that of the negative control pGL3-basic group (*p* < 0.01) but less than that of the pGL3-control group (*p* < 0.01).

## 4. Discussion

### 4.1. The Selection of Aggressive and Docile Pigs after Mixing

Pig behavioral traits are difficult to measure directly [29]. In our present study, aggressive behavior of weaned pigs after mixing was assessed according to the duration and frequency of active attack, being bullied and standoff behavior of pigs. Also, we used a calculated value CAS to select the two most aggressive and the two most docile pigs in each pen. Consistent differences of all behavioral indicators and CAS were found between the aggressive and docile pigs, which suggests that the selection of aggressive and docile pigs was valid.

### 4.2. Prediction and Identification of the Core Promoter in the Porcine MAOA Gene

Promoter is an important component of genes, a cis-acting element for the regulation of gene expression in eukaryotes, controlling the initiation location and expression abundance of gene expression [30]. In this study, the *MAOA* gene promoter region of pigs was identified and located at a CpG island (−692 bp to −585 bp), the CpG islands can influence local chromatin structure and regulate gene activity [31]. Furthermore, we predicted the binding sites for multiple transcription factors including AP-1, GATA3, CEBPA, and Sp1, which were well-known transcription factors involved in tumorigenesis and cell growth [32]. The core promoter region might not be only responsible for initiation of transcription [32], but it might play a role in tethering the elements that are required for basal activation of the *MAOA* gene.

### 4.3. Screening on SNPs in Porcine MAOA Gene Associated with Aggression

Previous studies have shown that aggressive behavior in pigs is moderately heritable [33], which indicates that it can be reduced through genetic selection [34]. In humans, reduced activity of the enzyme *MAOA* because of genetic polymorphisms within the *MAOA* gene leads to increased brain neurotransmitter levels associated with aggression [17]. Previous studies have showed that a VNTR (30 bp) in the promoter region of the *MAOA* gene affect its transcriptional activity [18]. Research on SNP rs1465108 of the *MAOA* gene in human also showed that the low functioning *MAOA* genotype heightened aggression [20]. We also found that the aggressive behavior of pigs after group mixing was related to the genetic polymorphism of the *MAOA* gene. The behavioral differences between genotypes indicate that the four linked SNPs in the *MAOA* gene were associated with pig aggressive behavior.

Low functioning *MAOA* genotypes have been reliably linked to increased reactive aggression [20]. For the four linked SNPs (rs321936011, rs331624976, rs346245147, and rs346324437), we found a consistent phenomenon that the wild genotypes (major allele frequency) were more aggressive than the mutant genotypes. This indicates that pigs with wild genotypes for the four linked SNPs probably had a lower level of *MAOA* expression compared to pigs with mutant genotypes. This is similar to a previous study in which *MAOA* minor allele was positively associated with reactive aggression [20]. The major genotypes of the four linked SNPs in the *MAOA* gene were expressed in high aggression, and minor genotypes were expressed in submissive behavior; the number of barrows were greater than the number of gilts in the major genotypes, which was consistent with the previous study that sex influenced the aggressive behavior in pigs [35]. The *MAOA* gene encodes monoamine oxidase A, which plays a crucial role in facilitating 5-HT degradation [13]. Our findings validate previous studies indicating that the *MAOA* major allele increases the likelihood of aggressive behavior in pigs.

### 4.4. In Vitro Expression of Different Genotypes in the Porcine MAOA Gene Promoter Region

Polymorphisms in the 5’ regulatory region of a gene could affect its transcriptional activity [36]. In the present study, luciferase reporter constructs with the GG genotype of rs321936011 had a lower promoter activity of the *MAOA* gene than that of AA genotypes. Enzyme *MAOA* degrades 5-HT and the concentration of 5-HT is positively related to aggression [15], which suggests that pigs with AA genotype of rs321936011 would be less aggressive. This is consistent with a previous study in human [20]. The SNP rs321936011 in the downstream of the core promoter region exerts an allele-specific effect on gene expression in vitro. The functional significance in expression supports the candidacy of *MAOA* as a candidate gene for pig aggression.

In the future studies, we may look the differences of DA and 5-HT in response to social stress in group-housed pigs. MAO-A functions as an enzyme involved in dopamine, norepinephrine, and serotonin metabolisms and related psychiatric disorders, including antisocial behavior [37]. The dysregulation of the DA and 5-HT systems have been found in humans and animals with aggressive behaviors [13].

## 5. Conclusions

The promoter activity analysis suggests that the core promoter region of the porcine *MAOA* gene is located between −679 bp and −400 bp from the initiation site of transcription. Four linked SNPs rs321936011, rs331624976, rs346245147, and rs346324437 located in the porcine *MAOA* gene were associated with aggressive behavior of pigs after group mixing. Pigs with wild genotypes (major allele frequency) in the *MAOA* gene were more aggressive than pigs with mutant genotypes (minor allele frequency). In vitro verification showed that the luciferase reporter constructs with GG genotype of rs321936011 had a lower promoter activity of *MAOA* gene than that of AA genotypes, which is consistent with the results of behavior assessment in vivo. These results indicate that the four linked SNPs in *MAOA* gene could be a candidate molecular marker for aggressive behavior in pigs.

## Figures and Tables

**Table 1:** Definitions of aggressive behavior traits used in the analyses.

Trait	Description
Fight	A vigorous biting, head-knocking, or a displacement with physical contact of two individuals for more than 3 s and intervening periods of at least 8 s, while the fight either was interrupted or the pigs showed other behaviors [22], including active attack, being bullied, and standoff.
Active Attack	In a fight, a pig give biting, pushing, chasing [23].
Being bullied	When the recipient pig suffers from biting and head-knocking performed by the actor pig the recipient moves away without retaliation, it was identified as a being bullied [24].
Standoff	If the two pigs stand side by side, shoulders by shoulders, and one pig throws his head to the head or neck of the other pig, there was an aggressive interaction with no dominance sign produced by either pair member at any time [25].
Win/lose/draw	A pig showing a submissive manner, such as stopping fighting, turning away from an attack, trying to flee or was displaced from the location, it was defined as a loser, the other pig in the fight was defined as a winner [26,27]. If there was no clear outcome, the fight was designated as a draw [22].

**Table 2:** The allele genotypes, frequencies, and significance of SNPs in the porcine *MAOA* gene between the most aggressive and the most docile pigs.

SNPs	Position	Region	SNP Site	Allele	Allele Frequency	MAF	HW P	χ^2^	*p*-Value
					(Aggressive/Docile Pigs)				
rs319522426	ChrX: 38,929,022	5’UTR	A > C	A	0.55/0.35	0.458	0.3672	8.083	0.004 **
(−1430 A or C)				C	0.45/0.65				
rs343493574	ChrX: 38,929,893	5’UTR	G > A	G	0.72/0.65	0.289	0.8452	1.038	0.308
(−559 G or A)				A	0.28/0.35				
rs321936011	ChrX: 38,930,422	5’UTR	G > A	G	0.75/0.65	0.295	0.6978	4.389	0.036 *
(−30 G or A)				A	0.25/0.35				
rs331624976	ChrX: 38,978,901	Intron 5	C > T	C	0.94/0.84	0.109	0.0268	10.046	0.002 **
(+48,450 C or T)				T	0.06/0.16				
rs346245147	ChrX: 38,993,909	Intron 11	A > G	A	0.63/0.47	0.425	0.9756	10.332	0.001 **
(+63,458 A or G)				G	0.37/0.53				
rs346324437	ChrX: 39,004,808	Intron 14	A > G	A	0.94/0.84	0.108	0.9197	9.813	0.002 **
(+74,357 A or G)				G	0.06/0.16				
rs336668628	ChrX: 39,006,600	3’UTR	A > G	A	0.84/0.81	0.184	0.7396	0.258	0.611
(+76,149 A or G)				G	0.16/0.19				
rs326512367	ChrX: 39,007,513	3’UTR	T > C	T	0.82/0.63	0.284	0.7494	8.841	0.003 **
(+77,062 T or C)				C	0.18/0.37				
rs325901151	ChrX: 39,008,156	3’UTR	A > G	A	0.88/0.79	0.152	0.0002	2.818	0.093
(+77,705 A or G)				G	0.12/0.21				

MAF: minor allele frequency; HW P: *p*-value of Hardy-Weinberg balance; χ^2^: Chi-square value; * *p* < 0.05, ** *p* < 0.01.

**Table 3:** Aggressive behavior indicators of pigs with different genotypes of the four linked SNPs.

SNP	Geno-Type	n (Barrows/Gilts)	CAS	Duration of Fights (s)	Frequency of Fights Initiated	Duration of Active Attacks (s)	Frequency of Active Attacks	Duration of Standoff (s)	Frequency of Standoff	Win
rs321936011	GG	114 (82/32)	4.32 ± 0.11 ^a^	7.11 ± 0.10 ^a^	3.00 ± 0.10	5.72 ± 0.11 ^a^	2.68 ± 0.10 ^ab^	6.54 ± 0.10 ^a^	2.28 ± 0.10 ^b^	2.51 ± 0.10
	AG	52 (1/51)	4.24 ± 0.17 ^a^	7.29 ± 0.17 ^a^	2.87 ± 0.17	5.52 ± 0.17 ^a^	2.97 ± 0.17 ^a^	6.99 ± 0.17 ^b^	2.88 ± 0.17 ^a^	2.62 ± 0.17
	AA	34 (21/13)	3.61 ± 0.17 ^b^	6.60 ± 0.17 ^b^	2.66 ± 0.17	4.86 ± 0.17 ^b^	2.38 ± 0.17 ^b^	5.88 ± 0.18 ^c^	2.04 ± 0.17 ^b^	2.21 ± 0.17
rs331624976	CC	168 (94/24)	4.26 ± 0.08 ^a^	7.15 ± 0.08 ^a^	2.97 ± 0.08	5.61 ± 0.08 ^a^	2.77 ± 0.08 ^a^	6.66 ± 0.08 ^a^	2.49 ± 0.08 ^a^	2.50 ± 0.08
	TC	20 (2/18)	4.13 ± 0.24 ^a^	6.90 ± 0.24 ^ab^	2.61 ± 0.23	5.49 ± 0.24 ^a^	2.63 ± 0.24 ^a^	6.24 ± 0.24 ^ab^	2.14 ± 0.24 ^ab^	2.61 ± 0.23
	TT	13 (8/5)	3.20 ± 0.28 ^b^	6.34 ± 0.28 ^b^	2.4 ± 0.28	4.54 ± 0.28 ^b^	1.72 ± 0.28 ^b^	5.65 ± 0.28 ^b^	1.69 ± 0.28 ^b^	2.25 ± 0.28
rs346245147	AA	83 (61/22)	4.51 ± 0.12 ^a^	7.31 ± 0.12 ^a^	3.17 ± 0.12 ^a^	5.89 ± 0.12 ^a^	2.92 ± 0.13 ^a^	6.78 ± 0.12 ^a^	2.50 ± 0.13	2.77 ± 0.12 ^a^
	GA	53 (5/48)	3.97 ± 0.16 ^b^	7.07 ± 0.16 ^ab^	2.68 ± 0.16 ^b^	5.26 ± 0.16 ^b^	2.70 ± 0.16 ^ab^	6.68 ± 0.16 ^a^	2.56 ± 0.16	2.33 ± 0.16 ^b^
	GG	63 (36/27)	3.88 ± 0.13 ^b^	6.76 ± 0.13 ^b^	2.68 ± 0.13 ^b^	5.24 ± 0.13 ^b^	2.38 ± 0.13 ^b^	6.17 ± 0.13 ^b^	2.19 ± 0.13	2.17 ± 0.13 ^b^
rs346324437	AA	168 (94/73)	4.26 ± 0.08 ^a^	7.15 ± 0.08 ^a^	2.97 ± 0.08 ^a^	5.61 ± 0.08 ^a^	2.77 ± 0.08 ^a^	6.67 ± 0.08 ^a^	2.49 ± 0.08 ^a^	2.49 ± 0.08 ^a^
	GA	22 (1/21)	4.05 ± 0.23 ^a^	6.83 ± 0.23 ^ab^	2.59 ± 0.23 ^ab^	5.41 ± 0.23 ^a^	2.54 ± 0.23 ^ab^	6.14 ± 0.23 ^ab^	2.08 ± 0.23 ^ab^	2.72 ± 0.23 ^a^
	GG	12 (9/3)	3.23 ± 0.29 ^b^	6.34 ± 0.29 ^b^	2.33 ± 0.29 ^b^	4.56 ± 0.29 ^b^	1.81 ± 0.29 ^b^	5.70 ± 0.29 ^b^	1.71 ± 0.29 ^b^	1.79 ± 0.29 ^b^

CAS, composite aggressive score = frequency of active attack + duration of active attack (s) * 0.2; the same superscript letter indicates no significant difference (*p* > 0.05); different superscript letters indicate statistically significant differences (*p* < 0.05).

**Table 4:** Aggressive behavior indicators of pigs with different haplotypes of the four linked SNPs.

Haplotypes	GCAA	ACGA	GCGA	ATGG	ACGG/GTAA
n (Barrows/Gilts)	82 (60/22)	55 (12/43)	29 (19/10)	22 (9/13)	6 (2/4)
Composite aggressive score	4.51 ± 0.12 ^a^	4.08 ± 0.15 ^b^	3.81 ± 0.19 ^bc^	3.46 ± 0.22 ^c^	3.95 ± 0.41 ^abc^
Duration of fights (s)	7.33 ± 0.12 ^a^	7.12 ± 0.14 ^ab^	6.66 ± 0.19 ^bc^	6.54 ± 0.22 ^c^	6.41 ± 0.41 ^bc^
Frequency of fights	3.62 ± 0.12 ^a^	3.49 ± 0.14 ^ab^	3.18 ± 0.19 ^b^	3.38 ± 0.21 ^ab^	2.86 ± 0.41 ^ab^
Frequency of fights initiated	3.15 ± 0.12 ^a^	2.91 ± 0.15 ^ab^	2.46 ± 0.19 ^b^	2.47 ± 0.22 ^b^	2.25 ± 0.41 ^b^
Duration of active attacks (s)	5.90 ± 0.12 ^a^	5.33 ± 0.15 ^b^	5.26 ± 0.20 ^bc^	4.82 ± 0.22 ^c^	5.34 ± 0.41 ^abc^
Frequency of active attacks	2.91 ± 0.12 ^a^	2.86 ± 0.15 ^a^	2.03 ± 0.19 ^bc^	1.92 ± 0.21 ^b^	2.22 ± 0.41 ^abc^
Frequency of being bullied	2.99 ± 0.12 ^ac^	3.16 ± 0.14 ^a^	2.54 ± 0.19 ^b^	3.05 ± 0.21 ^abc^	2.28 ± 0.41 ^bc^
Duration of standoff (s)	6.82 ± 0.12 ^a^	6.72 ± 0.14 ^a^	6.00 ± 0.19 ^b^	5.95 ± 0.21 ^b^	5.29 ± 0.41 ^b^
Frequency of standoff	2.51 ± 0.12 ^a^	2.73 ± 0.15 ^a^	1.88 ± 0.19 ^b^	1.81 ± 0.21 ^b^	1.58 ± 0.41 ^b^
Win	2.74 ± 0.12 ^a^	2.44 ± 0.14 ^a^	1.67 ± 0.19 ^b^	2.35 ± 0.22 ^a^	2.06 ± 0.41 ^ab^
Draw	1.36 ± 0.12 ^a^	1.39 ± 0.14 ^a^	1.67 ± 0.19 ^a^	0.22 ± 0.23 ^c^	−0.75 ± 0.42 ^b^

The same superscript letter in the same line indicates no significant difference (*p* > 0.05); different superscript letters in the same line indicate significant differences (*p* < 0.05).

## Supplementary Materials

The following are available online at https://www.mdpi.com/2076-2615/9/11/952/s1, Table S1: Primers for PCR in amplification of the MAOA gene.; Table S2: The numbers of barrows and gilts with different genotypes of four linked SNPs in the porcine MAOA gene; Table S3: The numbers of barrows and gilts with different haplotypes in the porcine MAOA gene; Table S4: Change of transcription factor-binding sites before and after the SNP rs321936011 mutation in the promoter region of the porcine MAOA gene.
