# Supplementary material for: Identification of Single Nucleotide Polymorphisms in Porcine MAOA Gene Associated with Aggressive Behavior of Weaned Pigs after Group Mixing
Source: Animals (Basel). 2019 Nov 11;9(11):952. doi: 10.3390/ani9110952 (PMC6912834; doi:10.3390/ani9110952)
Supplement: Supplementary file 1 [file animals-09-00952-s001.pdf]

animals

Supplementary Materials: Identification of Single Nucleotide Polymorphisms in Porcine *MAOA* Gene Associated with Aggressive Behavior of Weaned Pigs after Group Mixing

Ruonan Chen [1], Qingpo Chu [1], Chunyan Shen [1], Xian Tong [1], Siyuan Gao [1], Xinpeng Liu [1], Bo Zhou [1,*] and Allan P. Schinckel [2]

[1] College of Animal Science and Technology, Nanjing Agricultural University, Nanjing 210095, China; 2016105033@njau.edu.cn (R.C.); qpchu1990@163.com (Q.C.); 2016105082@njau.edu.cn (C.S.); 2017105081@njau.edu.cn (X.T.); 2018105082@njau.edu.cn (S.G.); 2018805122@njau.edu.cn (X.L.)

[2] Department of Animal Sciences, Purdue University, West Lafayette, IN 47907-2054, USA; aschinck@purdue.edu

* Correspondence: zhoubo@njau.edu.cn

Table S1. Primers for PCR in amplification of the *MAOA* gene.

Primer	Primer sequence (5'-3')	Product Size (bp)	Start	Stop
MAOA-1	F: CGTTTGCTTGTTTTCAACCT	412	-1 920	-1 901
	R: CTGGTGCTTTCATTGTTACG		-1 509	-1 490
MAOA-2	F: TTCCTTCAGTACGCGCT	621	-1 602	-1 586
	R: GAGTGTGTTACGTCTGGTG		-982	-964
MAOA-3	F: ATTCTGAGTGGCGGACT	504	-1 057	-1 041
	R: ATAACCCAAGTACTGACGCT		-554	-535
MAOA-4	F: GTCCCAGAAACTCTTAGACC	638	-699	-680
	R: GATTCTTCCATCGATACCCG		-62	-43
MAOA-5	F: TCAGTACAACAGTCCAGCCA	616	-480	-461
	R: ATCGATGCAGTCCACACTGA		135	154
MAOA-6	F: AGTCCAAGAGTCCAAGTGTGC	694	9 413	9 433
	R: GCTCAGATTCTACACACCAGC		10 106	10 086
MAOA-7	F: TGGTTCAGGTTGGTGGTTCA	405	18 511	18 530
	R: CAGGGCTACGGAGGACAAAT		18 915	18 896
MAOA-8	F: GCCTCACCAGTAACTAGGCAA	392	29 042	29 062
	R: AATGCCCTCCCATCAGTCACA		29 433	29 413
MAOA-9	F: GGTGGATTGGTTTTGGATCT	230	47 258	47 277
	R: ATCTGCTACTGATAATGCGG		47 487	47 468
MAOA-10	F: ATCCCAGACCCACCACTATCT	395	48 327	48 347
	R: CCTCCAAGGTTCTACTGATGG		48 721	48 701
MAOA-11	F: TGGAGTGGGATTGGCATGTT	431	53 265	53 284
	R: ACTGGGTGAGGGCAAAATCA		53 695	53 676
MAOA-12	F: GTATGGCCCGTGATTTTCTGG	388	56 736	56 756
	R: CAAGGCTGGTGATTCGGAAGA		57 123	57 103

MAOA-13	F: CCTCACAGTGGCTTCGTTCT	346	57 151	57 170
	R: CAAAGAGTGTGGTCCCGTCA		57 496	57 477
MAOA-14	F: GTCTGGGCGAACAAACATCTG	359	58 854	58 874
	R: TCCAAAACAACATGCTCTGCC		59 212	59 192
MAOA-15	F: GGTGTGGCCATAAAAATGAAT	450	63 231	63 251
	R: TCATGCAACAAGCACAATAG		63 680	63 661
MAOA-16	F: CATCGTGTGTAGTCACAGAA	258	68 535	68 554
	R: CTTAGAGCAAACAGAGCAGA		68 792	68 773
MAOA-17	F: AGCATTTTAGCTTTGTCTTCC	189	70 387	70 407
	R: CAGGTCTTGAATCGATTGGT		70 575	70 556
MAOA-18	F: AGTAAATGAGGGCTGAGATT	257	74 162	74 181
	R: GGAAAGATGAGATGCCAGTTT		74 418	74 398
MAOA-19	F: AGACCATGGTGACTTTCTTT	290	74 960	74 979
	R: ACAATGACATCAGGGCTAGA		75 249	75 230
MAOA-20	F: CCACATGCTGATTGTAGAGA	547	75 273	75 292
	R: ATCCTCAGGCGTTTTATGTT		75 819	75 800
MAOA-21	F: CTGGTGTTGATTGGTAGGAA	763	75 692	75 711
	R: TCTGCCCAATTTTACTGTGG		76 454	76 435
MAOA-22	F: TCACTCACTGAACATTCTCT	696	76 321	76 340
	R: AGGAGGTCTGTCAAAAGGTT		77 016	76 997
MAOA-23	F: AAACCTTTTGACAGACCTCC	533	76 996	77 015
	R: GGTATAAAATGCAGCAACCC		77 528	77 509
MAOA-24	F: GACCTTGCACTTGAAAAGAG	672	77 431	77 450
	R: AACTACACACAGTTCACGAT		78 102	78 083
MAOA-P1	F: CGAGCTCGGTATCGATGGAAGAATCGGC	216	-78	-59
	R: CCGCTCGAGCTATCGATGCAGTCCACACT		138	119
MAOA-P2	F: CGAGCTCGGTCCCATGAGGTGGTAAGAG	538	-400	-381
	R: CCGCTCGAGCTATCGATGCAGTCCACACT		138	119
MAOA-P3	F: CGAGCTCGCAATAACTTCCTACGAGGGC	817	-679	-660
	R: CCGCTCGAGCTATCGATGCAGTCCACACT		138	119
MAOA-P4	F: CGAGCTCGCGATAACTGACAAGGACATCA	1 076	-938	-918
	R: CCGCTCGAGCTATCGATGCAGTCCACACT		138	119
MAOA-P5	F: CGAGCTCGTTCAGCGTAACAATGAAAGC	1 672	-1 534	-1 515
	R: CCGCTCGAGCTATCGATGCAGTCCACACT		138	119
MAOA-P6	F: CGAGCTCGGGAGGAAATTTAAGGAAGGGA	2 286	-2 015	-1 996
	R: CCGCTCGAGCTATCGATGCAGTCCACACT		138	119
MAOA-P7	F: CGAGCTCGTCAGTACAACAGTCCAGCCA	616	-480	-461
	R: CCCAAGCTTATCGATGCAGTCCACACTGA		135	154

The base of the underlined marker is the cleavage site and the protective base.

Table S2. The numbers of barrows and gilts with different genotypes of four linked SNPs in the porcine *MAOA* gene.

SNP	Genotype	*n* (Male/Female)	χ2	*p*-value
rs321936011	GG	82/32	71.684	0.00 **
	AG	1/51		
	AA	21/13		
rs331624976	CC	94/24	15.648	0.00 **
	TC	2/18		
	TT	43682		
rs346245147	AA	61/22	54.407	0.00 **
	GA	5/48		
	GG	36/27		
rs346324437	AA	94/73	23.607	0.00 **
	GA	1/21		
	GG	9/3		

χ2: Chi-square value; ** $p < 0.01$.

Table S3. The numbers of barrows and gilts with different haplotypes in the porcine *MAOA* gene.

Haplotypes	*n* (Male/Female)	χ2	*p*-value
GCAA	60/22		
ACGA	12/43		
GCGA	19/10	38.857	0.00 **
ATGG	9/13		
ACGG/GTAA	2/4		

χ2: Chi-square value; ** $p < 0.01$.

Table S4. Change of transcription factor-binding sites before and after the SNP rs321936011 mutation in the promoter region of the porcine *MAOA* gene.

Transcription factor-binding sites	Score	Strand	Predicted site sequence	Appear (+) /Disappear (-)
EGR1	3.029	-1	TTTCTACCGACTCT	-
FOXC1	5.308	1	AGTCGGTA	-
FOXA1	1.044	-1	TTTCTATCGACTCTC	+
ZNF354C	4.828	-1	ATCGAC	+
